# Congenital basal meningoceles with different outcomes: a case series

**DOI:** 10.1186/s13256-017-1497-7

**Published:** 2017-12-27

**Authors:** Satomi Okano, Ryosuke Tanaka, Akie Okayama, Etsushi Tsuchida, Fumikatsu Nohara, Nao Suzuki, Toshio Okamoto, Ken Nagaya, Satoru Takahashi, Hiroshi Azuma

**Affiliations:** Department of Pediatrics, Asahikawa Medical University, 2-1-1-1 Midorigaoka-Higashi, Asahikawa, Hokkaido 078-8510 Japan

**Keywords:** Basal meningocele, Meningitis, Suction, Midfacial anomalies, Snore

## Abstract

**Background:**

Basal meningoceles are rare congenital defects and often clinically occult until they result in life-threatening complications. Therefore, it is important to know the diagnostic clues to early diagnosis.

**Case presentation:**

We describe three cases of congenital basal meningocele in a 3-year-old Japanese boy, a 1-month-old Japanese baby boy, and a 10-month-old Japanese baby girl. One of our patients died of sepsis due to traumatic rupture of the meningocele during nasal suction. His meningocele remained undiagnosed until it resulted in the fatal complication. The other patients underwent surgical repair without any complications. Their meningoceles were complicated by midfacial anomalies including cleft palate and hypertelorism, or a sign of nasal obstruction such as snoring.

**Conclusions:**

These clinical features may be a clue to the early diagnosis of congenital basal meningocele, which enables its safe preoperative management and provides an opportunity for surgical repair before the condition results in serious complications.

## Background

Basal meningoencephalocele is a rare congenital anomaly characterized by the herniation of brain tissue through the skull, with an incidence of 1 per 40,000 live births [1]. Southeast Asia is an endemic focus with an incidence of 1 per 6000 live births [2]. Compared with other encephaloceles, meningoceles contain meninges only and are classified according to the location of the bony defect: transethmoidal, sphenoethmoidal, transsphenoidal, spheno-orbital, and sphenomaxillary [3]. Congenital basal meningoceles are often clinically occult; however, a diagnosis makes it possible to perform the necessary surgical repair or prevent fatal episodes of meningitis. We report three cases of Japanese patients with congenital basal meningocele. In one of our patients, the basal meningocele remained undiagnosed and nasal suction caused traumatic cerebrospinal fluid (CSF) leaks resulting in fatal septic shock. The other patients underwent surgical repairs without any complications.

## Case presentation

### Patient 1

**Fig. 1:**
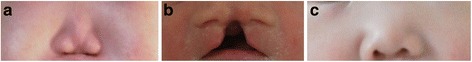
Facial appearance of the patients with congenital basal meningocele. All patients exhibited midfacial dysmorphism including hypertelorism and a broad nasal bridge. Patient 1 underwent surgical repair of cleft lip (**a**); patient 2 was born with cleft lip and palate (**b**); and patient 3 had a slightly warped left ala nasi (**c**)

Our patient was a 3-year-old Japanese boy who was born to non-consanguineous parents. A prenatal ultrasonographic examination revealed hydrocephalus and midline cleft lip. After birth he was found to have median cleft lip and palate, atresia of the anus, and micropenis. He also had facial dysmorphism including hypertelorism and a saddle nose (Fig. 1a). Brain magnetic resonance imaging (MRI) revealed agenesis of the corpus callosum and gyral malformations. Chromosomal analysis revealed a normal karyotype. His psychomotor development was severely delayed, including the absence of head control, eye pursuit, or the use of meaningful words. He required tracheostomy and 24-hour ventilation assistance owing to refractory epilepsy and repeated aspiration pneumonia. Because of feeding problems resulting from difficulties in swallowing, he was fed through a gastrostomy tube.

**Fig. 2:**
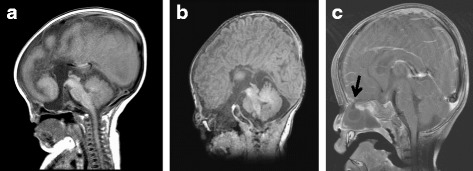
Brain magnetic resonance imaging scans of the patients with congenital basal meningocele. Sagittal T1-weighted images indicated transethmoidal meningocele and agenesis of the corpus callosum in patient 1 (**a**), sphenoethmoidal meningocele and agenesis of the corpus callosum in patient 2 (**b**), and transethmoidal meningocele (arrow) in patient 3 (**c**)

His mother performed nasal suction at home, and shortly thereafter found continuous discharge of clear water from one side of his nose. He was transferred to our hospital. At admission, he was in shock and comatose. His white blood cell count of 18,600/μL and C-reactive protein level of 6.28 mg/dL indicated bacterial infection. He was treated with meropenem and gamma globulin. Vancomycin was started until *Streptococcus pneumoniae* was isolated from blood culture. A computed tomography scan of his head demonstrated narrow ventricles, intracranial hemorrhage, and air in the lateral ventricle, suggesting the presence of a communication between the intracranial and extracranial cavities. An ethmoid defect and a frontoethmoidal meningocele were diagnosed on the basis of a retrospective review of the initial MRI (Fig. 2a). Thus, CSF rhinorrhea resulted from a traumatic rupture of the meningocele. He died on the 26th day of hospitalization because of intestinal necrosis and perforation that probably resulted from septic shock.

### Patient 2

Our patient was a 1-month-old Japanese baby boy who is the first child of healthy, unrelated parents. He was born through an emergency caesarian section because of obstructed labor at full term. His birth weight was 2856 g and head circumference was 36.3 cm (>90th percentile). His Apgar score was 8 at 1 minute and 9 at 5 minutes; however, respiratory distress became obvious because a meningeal sac protruding through a wide cleft palate obstructed his oral cavity. Although he was intubated immediately in the operation room, he needed no more respiratory support; thus, he was extubated on admission to our neonatal intensive care unit.

A physical examination revealed hypertelorism, a broad nasal bridge, midline cleft of the palate and upper lip, and V-shaped forehead hairline (Fig. 1b). This phenotype was compatible with frontonasal dysplasia [4]. MRI demonstrated that a sphenoethmoidal meningocele protruded through a wide cleft palate and occupied his oral cavity (Fig. 2b). Laboratory tests including hormonal assessments were normal. His karyotype was 46,XY. Milk was fed through a nasogastric tube, and moisturizing gel was applied on the meningocele to prevent rupture. Surgical repair was successfully done at the age of 45 days. The meningocele was lifted into intracranial space and stitched on the cranial base closing the defect. Sutured dura matter was reinforced with free muscle flap from right temporalis. His pituitary gland was normal. He could drink milk well and was discharged at the age of 64 days.

### Patient 3

A 10-month-old Japanese baby girl was referred to our hospital because of loud snoring. Her growth and developmental milestones were normal. A physical examination revealed hypertelorism, a slightly warped ala nasi, and a soft tissue mass in her left nasal cavity (Fig. 1c). MRI demonstrated a transethmoidal meningocele protruding into the left nasal cavity (Fig. 2c). Surgical repair was successfully done 2 months later. Skull defect repair was done using periosteum and patched with endocranium of the cerebral falx. We confirmed that the left basal skull bone had dropped downward and the left olfactory nerve was missing. After the operation, her snoring disappeared.

## Discussion

We reported three contrasting cases of congenital basal meningocele. The first case exhibited multiple congenital anomalies, and the meningocele remained undiagnosed until it resulted in fatal sepsis due to traumatic rupture. His watery rhinorrhea and intracranial air suggested meningitis although we could not collect spinal fluid. Meningitis is well known to be a serious complication of basal meningoceles [5] and can certainly cause death. In reality, few cases of death due to basal meningocele meningitis are reported. We searched the literature in PubMed with the terms “meningocele meningitis” (235 cases) and “basal meningocele meningitis” (8 cases); however, we could not find any patients with congenital basal meningocele who had died of meningitis except for surgical complications [6]. Our first case suggests the importance of early diagnosis and careful preoperative management of congenital basal meningoceles for such children.

Congenital basal meningoceles are often clinically occult until they result in life-threatening complications. Therefore, it is important to know the diagnostic clues to early diagnosis. All cases exhibited facial anomalies including hypertelorism and a broad nasal bridge. Two of our patients further exhibited cleft lip and palate. It is important to note that basal meningoencephalocele is often complicated by such facial dysmorphisms [7, 8]. Thus, facial midline deformities may suggest the possible defect of a skull base, and should alert clinicians about an unrecognized meningocele. In our third case, snoring was a sign of airway obstruction due to the meningocele. Noisy breathing sounds have been reported as a clue to the diagnosis [9, 10].

## Conclusions

Our cases emphasize the potential risks of basal meningoceles. Early diagnosis of occult basal meningoceles enables their safe preoperative management and provides an opportunity for surgical repair before they result in serious complications. Facial midline deformities and signs of upper airway obstruction may be diagnostic clues to congenital basal meningoceles.

## Abbreviations

CSF: Cerebrospinal fluid
MRI: Magnetic resonance imaging
